# Robust Controller Design for Multi-Input Multi-Output Systems Using Coefficient Diagram Method

**DOI:** 10.3390/e23091180

**Published:** 2021-09-08

**Authors:** Kai Liu, Fanwei Meng, Shengya Meng, Chonghui Wang

**Affiliations:** College of Department of Control Science and Engineering, Northeastern University at Qinhuangdao, Qinhuangdao 066004, China; miaomiao@stumail.neu.edu.cn (K.L.); 2001944@stu.neu.edu.cn (S.M.); 2071918@stu.neu.edu.cn (C.W.)

**Keywords:** MIMO, coupling, PSO, CDM, measurement noise, robust controller

## Abstract

The coupling between variables in the multi-input multi-output (MIMO) systems brings difficulties to the design of the controller. Aiming at this problem, this paper combines the particle swarm optimization (PSO) with the coefficient diagram method (CDM) and proposes a robust controller design strategy for the MIMO systems. The decoupling problem is transformed into a compensator parameter optimization problem, and PSO optimizes the compensator parameters to reduce the coupling effect in the MIMO systems. For the MIMO system with measurement noise, the effectiveness of CDM in processing measurement noise is analyzed. This paper gives the control design steps of the MIMO systems. Finally, simulation experiments of four typical MIMO systems demonstrate the effectiveness of the proposed method.

## 1. Introduction

Multi-input multi-output (MIMO) systems, defined as systems with multiple control inputs and outputs, are widely used in industrial systems. Many common industrial control systems can be modeled as MIMO systems, such as chemical reactors, distillers, generators, and automobile transmission systems [1,2,3,4,5]. A consensus is that the control of the MIMO systems is more complex than the control of the single-input single-output (SISO) systems. In the MIMO systems, the outputs are affected by each input. In other words, there is a coupled interaction between the input and output variables of the MIMO systems. Due to the interaction in the MIMO systems, it is not easy to directly apply the advanced control methods based on the SISO systems.

Currently, the control strategies of the MIMO systems are mainly based on the methods of decoupling. Decoupling strategies can be divided into static decoupling and dynamic decoupling. The former achieves decoupling based on steady-state gain, which can effectively reduce the impact of model uncertainty, but the high-frequency response of MIMO systems is often not ideal [6]. The dynamic decoupling can achieve a trade-off between complexity and decoupling performance. In recent years, various dynamic decoupling strategies have been developed, such as ideal decoupling, simplified decoupling, and reverse decoupling. The ideal decoupler can provide a simple decoupling system, but the ideal decoupler is difficult to realize in practical applications. The opposite is simplified decoupling. Although simplified decoupling can obtain simple decoupling and decoupling, the decoupling system will be very complicated. In [7], Hagglund T proposed a decoupling method that approximates the sum of elements to reduce the system’s complexity after decoupling. Reverse decoupling takes into account the advantages of ideal decoupling and simplified decoupling. However, when there is a time-delay element in the MIMO systems, reverse decoupling cannot guarantee the system’s stability. In addition, researchers have used intelligent algorithms for MIMO systems and proposed various intelligent decoupling algorithms [8,9,10,11,12]. However, because the design of this kind of method is difficult to understand and the controller is complicated, it is difficult for engineers to adopt.

As an algebraic design method, the CDM proposed by S. Manabe is simple and easy to implement [13]. Compared with other control methods, CDM only requires the designer to define one parameter: the equivalent time constant [14]. At the same time, all algebraic equations in the CDM are expressed in the form of polynomials, which facilitates the elimination of poles and zeros in the design and analysis of the control systems. CDM has been proven to be a method to ensure the robustness of the control system, and its effectiveness has been proven through a series of experiments [15,16,17]. Therefore, with the continuous improvement of CDM, CDM has been continuously applied to existing control systems. Mohamed T. H. combined CDM with ecological optimization technology (ECO) for load frequency design in multi-regional power systems in [18]. Experimental results show that the proposed method is robust in the presence of disturbance uncertainty. Because CDM is simple, effective and robust, it is also applied in MIMO system control [19]. CDM was be used to design a PI controller with two cone-shaped official position research objects in [20]. The simulation results prove the effectiveness of CDM on disturbance suppression. In [21], CDM was used to solve the controller gain to suppress the vibration in the flexible robot system.

The first problem to be solved in the controller design of the MIMO system is how to achieve decoupling. Compared with other existing results, this article transforms the decoupling problem into the parameter optimization problem and gives an interaction measurement to evaluate the decoupling degree of the MIMO systems. The PSO algorithm is used to optimize the parameters of the compensator to achieve decoupling. After decoupling, the systems tend to have high order. The CDM considers the robustness and interference suppression performance of the system and the simplicity of design. Therefore, motivated by the advantages of CDM, this paper applies the CDM to the field of controller design for MIMO systems. At the same time, considering measurement noise can generate undesired control activity resulting in wear of actuators and reduced performance, this article analyzes the controller’s suppression effect on measurement noise based on the CDM. To verify the effectiveness and universality of the proposed method, this paper gives four typical design examples of MIMO systems in the hope of providing engineers and technicians a reference.

The main innovations of this paper are as follows:

(1) Converts the compensator design problem used for decoupling into parameter optimization problems to reduce the difficulty of decoupling.
(2) Gives an interaction measurement to quantify the interaction of coupled systems.
(3) Analyzes the controller’s suppression effect on measurement noise based on the CDM.
(4) Research on the application of CDM methods to MIMO systems needs to be promoted. In order to make up for the shortcomings of existing research, this paper presents a design strategy of a robust controller based on CDM, which provides a reference for the design of the MIMO system controller in other articles.

The rest of this article is organized as follows: Section 2 gives an interaction measurement of the coupling interaction and uses the PSO algorithm to design the compensator to achieve decoupling; Section 3 summarizes the design process of the CDM controller and analyzes the controller’s suppression effect on measurement noise based on the CDM. Section 4 outlines a set of controller design procedures for MIMO systems; four unique objects are simulated to verify the effectiveness of the proposed method in Section 5. Finally, a conclusion is given.

## 2. Decoupling Design

At present, there are two solutions to the interaction of MIMO systems. One is to use modern control theory, and the other is to limit the interaction to a certain extent and treat MIMO systems as multiple SISO systems, which is called decoupling control. Generally speaking, decoupling control is simple to operate, so it is often used. This article designs a compensator in the frequency domain to decouple. At the same time, in order to verify whether the designed compensator achieves the expected decoupling effect, this section provides the MIMO system interaction measurement.

### 2.1. Compensator Design

**Figure 1:**
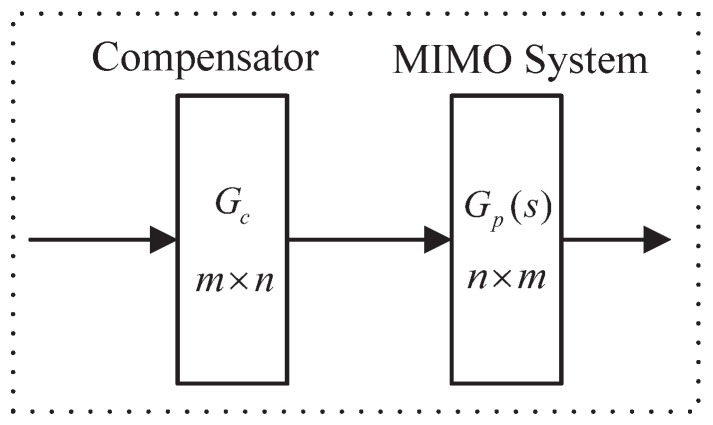
Schematic diagram of the decoupling design of the n×m MIMO system.

The schematic diagram of the decoupling design of the n×m MIMO system is shown in Figure 1.

where the model of the MIMO system is represented by the transfer function $G_{p}\left(s\right)\in R^{n\times m}$, which is Equation (1).

(1) $$\begin{array}{c} G_{p}\left(s\right)=\left[\begin{array}{ccc} g_{11}\left(s\right) & \cdots & g_{1m}\left(s\right)\\ \cdots & \ddots & \cdots \\ g_{n1}\left(s\right) & \cdots & g_{nm}\left(s\right) \end{array}\right]. \end{array}$$

where $g_{ij}\left(s\right),i=1,2,\cdots ,n;j=1,2,\cdots ,m$ is the transfer function element in $G_{p}\left(s\right)$. Design the compensator $G_{c}\left(s\right)\in R^{m\times n}$ as shown in Equation (2).

(2) $$\begin{array}{c} G_{c}\left(s\right)=\left[\begin{array}{ccc} h_{11}\left(s\right) & \cdots & h_{1n}\left(s\right)\\ \cdots & \ddots & \cdots \\ h_{m1}\left(s\right) & \cdots & h_{mn}\left(s\right) \end{array}\right], \end{array}$$

where $h_{ij}\left(s\right)\left(i=1,2,\cdots ,m;j=1,2,\cdots ,n\right)$ is the transfer function element in $G_{c}\left(s\right)$. In order to reduce the difficulty of the designed compensator $G_{c}\left(s\right)$, set $G_{c}\left(s\right)$ as a constant matrix. When the compensator $G_{c}$ acts on the MIMO system $G_{p}\left(s\right)$, the decoupling system $Q\left(s\right)\in R^{n\times n}$ is obtained as Equation (3).

(3) $$\begin{array}{c} \begin{array}{c} Q\left(s\right)=G_{p}\left(s\right)G_{c}\\ \begin{array}{cc} \begin{array}{ccc} & & \;\;\;\;=\;\;\;\;\;\; \end{array} & \left[\begin{array}{ccc} g_{11}\left(s\right) & \cdots & g_{1m}\left(s\right)\\ \ldots & \ddots & \ldots \\ g_{n1}\left(s\right) & \ldots & g_{nm}\left(s\right) \end{array}\right] \end{array}\left[\begin{array}{ccc} h_{11} & \cdots & h_{1n}\\ \ldots & \ddots & \ldots \\ h_{m1} & \cdots & h_{mn} \end{array}\right]\\ \begin{array}{ccc} & & \;=\;\;\left[\begin{array}{ccc} f_{11}\left(s\right) & \cdots & f_{1n}\left(s\right)\\ \cdots & \ddots & \cdots \\ f_{n1}\left(s\right) & \cdots & f_{nn}\left(s\right) \end{array}\right]. \end{array} \end{array} \end{array}$$

The purpose of designing the compensator is to make the decoupling system Q(s) diagonal in all frequency domains, which means non-diagonal elements $f_{lr}\left(s\right)=0$ (l≠r, l=1,2,⋯n, r=1,2,⋯n). In this way, the interaction is minimized, and the decoupling effect is the best. However, it is not easy to find such an ideal compensator. Therefore, this paper selects a specific frequency $s=j\omega_{0}$ to design the compensator. What needs to be explained is that the selection of a specific frequency $s=j\omega_{0}$ depends on the control object and requires the designer to use design experience to verify it through repeated experiments.

Use $s=j\omega_{0}$ to denote the element in the *r*(r=1,2,⋯n) column of $Q\left(s\right)=G_{p}\left(s\right)G_{c}$, we can get

(4) $$\begin{array}{c} \begin{array}{c} f_{lr}\left(j\omega_{0}\right)=g_{l}\left(j\omega_{0}\right){\hat{h}}_{r}=(\alpha_{l}+j\beta_{l}){\hat{h}}_{r}\;\;\;\;\;\;l=1,\;2,\;\cdots n, \end{array} \end{array}$$

where $g_{l}\left(j\omega_{0}\right)$ is the $G_{p}\left(j\omega_{0}\right)$ row vector of *l*, ${\hat{h}}_{r}$ is the $G_{c}$ column column vector of *r*, αl=Regl(jω0), and βl=Imgl(jω0).

In order to achieve $Q(j\omega_{0})$ diagonalization, we make the absolute value square of the off-diagonal elements in the *r*(r=1,2,⋯n) column of $Q(j\omega_{0})$ equal to zero, which is

(5) $$\begin{array}{c} {\left|f_{lr}\left(j\omega_{0}\right)\right|}^{2}={\hat{h}}_{r}^{T}\left(\alpha_{l}{\alpha_{l}}^{T}+\beta_{l}{\beta_{l}}^{T}\right){\hat{h}}_{r}=0\;\;\;\;\;\;l\neq r. \end{array}$$

Under Equation (5), the optimal solution ${\hat{h}}_{r}$ can be obtained, thereby obtaining the compensator $G_{c}$ and the decoupling system Q(s). However, the decoupling system Q(s) may not meet the decoupling design requirements. One reason is that under the condition of a certain frequency $s=j\omega_{0}$, Equation (5) can only guarantee that the absolute value square of the off-diagonal elements of $Q(j\omega_{0})$ is equal to zero, but the absolute value square of diagonal elements ${\left|f_{lr}\left(j\omega_{0}\right)\right|}^{2}$(l=r) is not equal to zero or does not tend to zero. Suppose the designed compensator $G_{c}$ cannot guarantee that Q(s) at a certain frequency $s=j\omega_{0}$ achieves diagonalization. In that case, there is no guarantee that Q(s) can be decoupled in the entire frequency domain. The other reason is that the ${\hat{h}}_{r}$(r=1,2,⋯n) may be a trivial solution, so the compensator designed is meaningless. To effectively illustrate the above description, we give a concrete example next.

**Example** **1.** *Consider the two-input two-output one-order inertial system, The transfer function is:*

(6) $$G_{p}=\left[\begin{array}{cc} \frac{2}{6s+1} & \frac{-3.6}{4s+1}\;\\ \frac{0.4}{9s+1} & \frac{4}{42s+1} \end{array}\right].$$

*we select the frequency $\omega_{0}=1$, and under Equation (5), use PSO to obtain the compensator as equation:*

(7) $$\begin{array}{ccc} & & G_{c}=\left[\begin{array}{cccc} -1.2499\times 10^{-16} & & 3.2508\times 10^{-22} & \\ 1.7313\times 10^{-17} & & 1.0364\times 10^{-21} \end{array}\right]. \end{array}$$

It can be seen from Equation (7) that without any restrictions, the calculated $G_{c}$ is meaningless. Therefore, taking into account the above deficiencies, we make the following additions based on the constraint condition of Equation (5): Firstly, we select the square ${\left|f_{lr}\left(j\omega_{0}\right)\right|}^{2}$ (l=r) of the absolute value of diagonal elements of $Q(j\omega_{0})$*r*(r=1,2,⋯n) column as the objective function to obtain its maximum value. Secondly, in order to prevent the trivial solution of the obtained ${\hat{h}}_{r}$, we add the Equation (8) as the constraint condition:

(8) $$\begin{array}{c} {\hat{h}}_{r}^{T}{\hat{h}}_{r}=1. \end{array}$$

In summary, the decoupling problem of MIMO systems is transformed into the optimization problem as follows:

(9) $$\begin{array}{c} \begin{array}{c} \max\;f({\hat{h}}_{r})={\hat{h}}_{r}^{T}\left(\alpha_{l}{\alpha_{l}}^{T}+\beta_{l}{\beta_{l}}^{T}\right){\hat{h}}_{r}\;\;\;\;\;l=r,\\ s.t.\;\;{\hat{h}}_{r}^{T}\left(\alpha_{l}{\alpha_{l}}^{T}+\beta_{l}{\beta_{l}}^{T}\right){\hat{h}}_{r}=0\;\;\;\;\;l\neq r,\\ \;\;\;\;\;\;{\hat{h}}_{r}^{T}{\hat{h}}_{r}=1. \end{array} \end{array}$$

### 2.2. Interaction Measurement

The design process of the compensator has been given in Section 2.1. Since the magnitude of the interaction between the variables of the decoupling system Q(s) does not have a specific numerical measurement, it is not clear whether the designed compensator can achieve the desired decoupling effect. Therefore, this section presents an interaction measurement for the MIMO system to evaluate the impact of the decoupling degree of the compensator. The equation is established on the basis that the diagonal elements of the diagonal matrix are equal to the reciprocal of the diagonal elements of its inverse.

Assuming that the controlled variable of the decoupling system Q(s) is $Y={\left[y_{1},y_{2},\cdots ,y_{n}\right]}^{T}$, the manipulated variable is $U={\left[u_{1},u_{2},\cdots ,u_{n}\right]}^{T}$, and $u_{i}$ (i=1⋯n) controls $y_{i}$. For the i-th channel of Q(s), the open-loop gain of the channel is obtained when all other manipulated variables are zero, that is, equality (10). The open-loop gain of the channel is obtained when all other controlled variables are zero, that is, equality (11).

(10) $$\begin{array}{c} \mathrm{other}\;\mathrm{loops}\;\mathrm{are}\;\mathrm{open}:{\left(\frac{\partial y_{i}}{\partial u_{i}}\right)}_{u_{n}=0,n\neq i}=f_{ii}. \end{array}$$

(11) $$\begin{array}{c} \mathrm{other}\;\mathrm{loops}\;\mathrm{are}\;\mathrm{closed}:{\left(\frac{\partial y_{i}}{\partial u_{i}}\right)}_{y_{n}=0,n\neq i}={\tilde{f}}_{ii}. \end{array}$$

Here, measurement for Q(s) interaction in MIMO systems is given:

(12) $$\begin{array}{c} E=\sum_{i=1}^{n}\frac{\left|f_{ii}-{\tilde{f}}_{ii}\right|}{\left|{\tilde{f}}_{ii}\right|}\;\;\;i=1∼n. \end{array}$$

When the decoupling system Q(s) is diagonalized, E=0. Therefore, when Equation (12) is equal to zero or close to zero, it shows that other channels have no or minimal relationship with the channel, and the decoupling effect is good.

**Remark** **1.** *Equations (10) and (11) are based on the steady-state of the MIMO system, but this situation is usually not maintained at other frequencies. Therefore, it can only be used as the measurement of the interaction size of the MIMO system and cannot be used as the judgment of whether the MIMO systems are decoupled.*

### 2.3. Parameter Tuning of Compensator

In this paper, particle swarm optimization (PSO) is used to optimize the objective function. Firstly, the fitness function is compiled. In order to facilitate programming, $-{\left|f_{lr}\left(j\omega_{0}\right)\right|}^{2}$ ( l=r ) is taken as the objective function to obtain its minimum value. The fitness function can be obtained as follows:

(13) $$\begin{array}{c} \begin{array}{c} Fit\left[f\left({\hat{h}}_{r}\right)\right]=-{\left|f_{lr}\left(j\omega_{0}\right)\right|}^{2}=-{\hat{h}}_{r}^{T}\left(\alpha_{l}{\alpha_{l}}^{T}+\beta_{l}{\beta_{l}}^{T}\right){\hat{h}}_{r}\;\;\;\;\;\;l=r. \end{array} \end{array}$$

**Figure 2:**
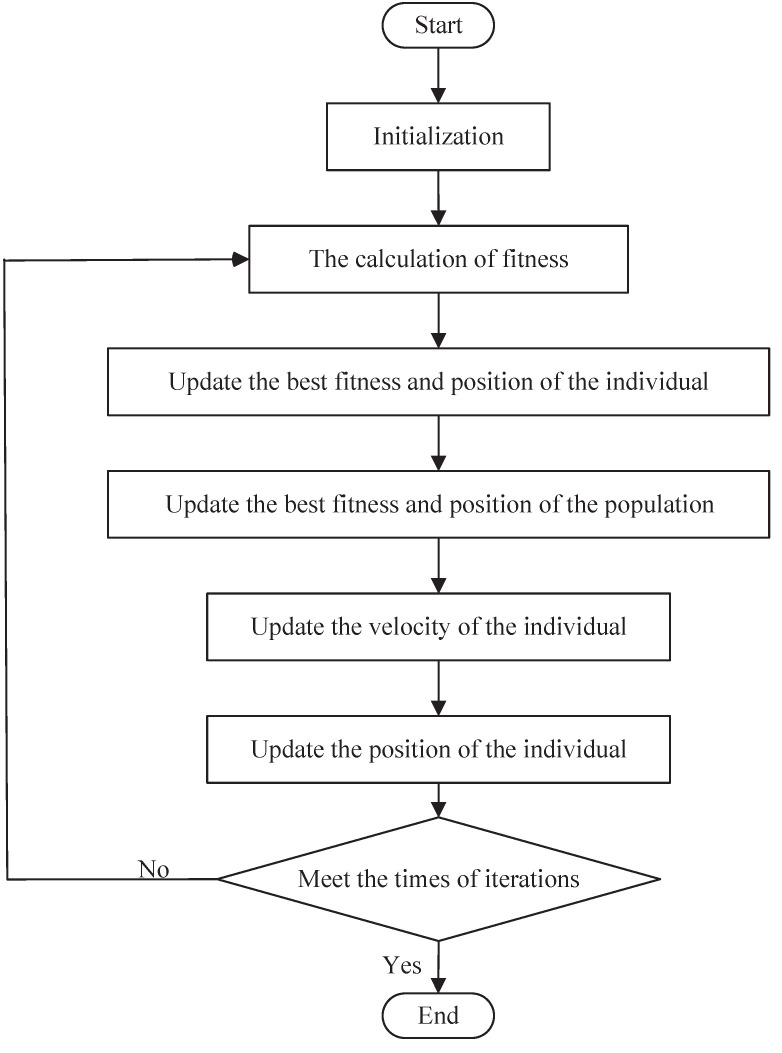
The flowchart of PSO.

Secondly, through the above fitness function and constraints in the frequency domain, ${\hat{h}}_{r}$ ( r=1,2,⋯n ) can be obtained through PSO debugging, and thus the compensator $G_{c}$ can be obtained. Figure 2 shows the flowchart of PSO.

PSO is essentially a stochastic algorithm, which has the function of self-organization, evolution, and memory and the strong searching ability and fast optimizing speed. In order to demonstrate the superiority of PSO to other evolutionary algorithms, we execute a number of comparisons between PSO and other evolutionary algorithms, such as Genetic Algorithm (GA), Shuffled Frog Leaping Algorithm (SFLA) and Cuck Search (CS). GA originates from Darwin’s idea of natural evolution and follows the natural law of competition and survival of the fittest. GA is characterized by fast search speed, strong randomness, simple process, and robust flexibility. Still, it is easy to fall into the local optimum due to the reduction of population diversity in the evolution process. CS is a new swarm intelligence algorithm based on simulating cuckoo’s nesting behavior. The algorithm has been successfully applied to solve various optimization problems due to its fewer parameters and easy realization. A significant feature of the CS is that it uses Levy flight to generate new solutions. The high randomness of Levy flight is that it can make the search process throughout the whole search space so that the global search ability of the algorithm is strong. However, the Levy flight height’s randomness causes the CS algorithm’s poor ability to perform a refined search in the local area and the slow convergence of the algorithm. SFLA simulates the communication and cooperation behaviors of frog populations in the process of foraging in nature, which has the advantages of fewer control parameters, simple operation, and easy realization. The specific parameter settings of different evolutionary algorithms are proposed in Table 1. The population size of each algorithm is 50, and the times of iterations are 100. The crossover probability and mutation probability of GA are 0.9 and 0.1, respectively. SFLA’s moving maximum distance is 0.02, CS’s maximum discovery probability is 0.05. The weight of inertia, the self-learning factor and the population-learning factor of PSO are 0.35, 1.5 and 2.5. respectively.

**Figure 3:**
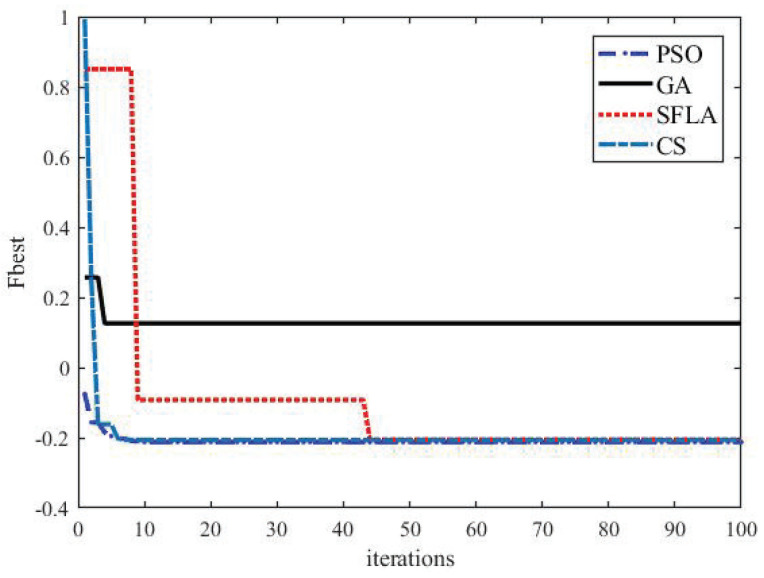
Convergence graphs of the optimization algorithms.

In order to facilitate a comparison, we randomly select $G_{p}\left(s\right)\;=\;\left[0.2,0.5;-0.3,0.6\right]$, set $G_{c}\;=\;\left[h_{1},h_{2};h_{3},h_{4}\right]$, and only obtain the first column ${\hat{h}}_{1}$ : $h_{1}$ and $h_{3}$ of $G_{c}$. Each algorithm is implemented independently 30 times. Table 2 presents the statistical results of each algorithm, including the maximum, minimum, average, standard deviation values of the objective function, and the average computational time. According to Table 2, we can see that PSO has an evident advantage of minimum, average, standard deviation values and average computational time over other algorithms. Figure 3 is the convergence graph of the optimization algorithms. It can be seen that PSO has a fast convergence speed and a good effect in finding the optimal global solution.

Furthermore, according to the research works with respect to the non-parametric statistical tests for different algorithms [22], some statistical tests have been adopted to compare the performance of GA, SFLA, CS and PSO. Table 3 proposes ranks achieved by Friedman, Friedman aligned and Quade tests for the objective function obtained by different algorithms. It is noticeable from Table 3 that PSO performs best in all statistical tests. Consequently, PSO has the superiority over other evolutionary algorithms in solving unknown parameters of compensator $G_{c}$.

## 3. CDM Controller Design and Measurement Noise Rejection

In Section 2, the decoupling design can obtain the decoupling system Q(s) with minimized interaction, but its open-loop transfer function is complex, and the order is high. Therefore, when stability, response characteristics, and robustness are considered simultaneously, the designed controller will become more complicated. CDM can effectively solve such problems.

### 3.1. CDM Controller Design

**Figure 4:**
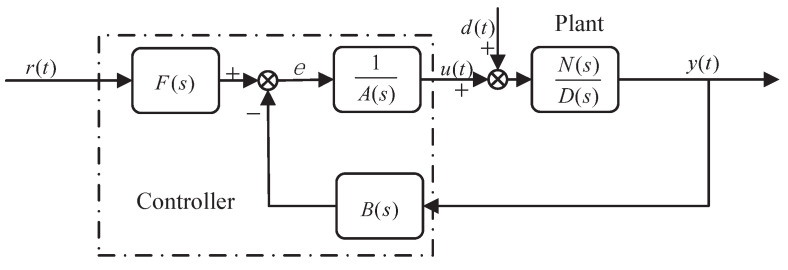
CDM control system standard block diagram.

For the SISO linear systems, the standard block diagram designed by CDM is shown in Figure 4. The CDM control system consists of two parts: the controlled object and the CDM controller.

where r(t), u(t), y(t) and d(t) are reference signal, control quantity, output quantity and disturbance quantity, respectively. The control function of the controller u(t) may be interfered by the interference signal *d*. N(s) and D(s) are the numerator and denominator polynomials of the controlled object, respectively, defined as follows:

(14) $$\begin{array}{c} \begin{array}{c} N\left(s\right)=b_{m}s^{m}+b_{m-1}s^{m-1}+\cdots +b_{1}s+b_{0},\\ D\left(s\right)=d_{n}s^{n}+d_{n-1}s^{n-1}+\cdots +d_{1}s+d_{0}, \end{array} \end{array}$$

where $b_{m},b_{m-1}\cdots b_{0}$ and $d_{n},d_{n-1}\cdots d_{0}$ are real coefficients and m≤n. A(s) and B(s) are the denominator and numerator polynomial of the controller, respectively, defined as follows:

(15) $$\begin{array}{c} A\left(s\right)=\sum_{i=0}^{p}l_{i}s^{i},\;\;\;\;B\left(s\right)=\sum_{i=0}^{q}k_{i}s^{i}, \end{array}$$

where $l_{i}$ and $k_{i}$ are unknown coefficients of the controller and i≤n. There are many criteria for the selection of A(s) and B(s) polynomials. Disturbance is one of the selection criteria. When $l_{0}=0$, the influence of disturbance signal can be well suppressed. F(s) is the reference numerator of the controller, which can ensure that the steady-state error in the performance of the closed-loop system is reduced to zero. The definition form is as follows:

(16) $$\begin{array}{c} F\left(s\right)={\left(\frac{P(s)}{N(s)}\right)}_{|s=0}, \end{array}$$

where P(s) is the characteristic polynomial of the closed-loop system. From Figure 4, we can obtained

(17) $$\begin{array}{c} \begin{array}{c} P\left(s\right)=D\left(s\right)A\left(s\right)+N\left(s\right)B\left(s\right)=\sum_{i=0}^{n}a_{i}s^{i},a_{i}>0, \end{array} \end{array}$$

where $a_{i}$ is the real coefficient. The design parameters of CDM-related characteristic polynomials are equivalent to the time constant τ and stability index $\gamma_{i}$, defined as follows:

(18) $$\begin{array}{c} \left\{\begin{array}{c} \tau =\frac{a_{1}}{a_{0}},\\ \gamma_{i}=\frac{{a_{i}}^{2}}{a_{i+1}a_{i-1}}\begin{array}{cc} , & i=1∼n-1, \end{array}\\ \gamma_{0}=\gamma_{n}=\infty ,\\ {\gamma_{i}}^{*}=\frac{1}{\gamma_{i+1}}+\frac{1}{\gamma_{i-1}}, \end{array}\right. \end{array}$$

where ${\gamma_{i}}^{*}$ denotes the stability limit, which is used to constrain the value of the stability index $\gamma_{i}$, and ${\gamma_{i}}^{*}$ is mainly used to ensure that it meets the Lyapunov stability conditions in the actual design process. The equivalent time constant τ is closely related to the setting time and bandwidth, which determines the rapid response of the system. If the setting time is represented by $t_{s}$, according to the Manabe standard form [13], its relationship with the equivalent time constant $t_{s}$ is τ=tsts(2.5∼3)(2.5∼3).

The selection of the stability index $\gamma_{i}$ determines the stability and time domain response characteristics of the system. Robustness is different from the system’s stability, mainly considering the influence of system parameters on the speed of pole change. Control systems with other structures may have different robustness even if they have the same characteristic equation. The robustness of the system can only be determined when the open-loop system structure is determined. An essential feature of CDM in the application is that the controller structure and the characteristic polynomial can be designed simultaneously, and the robustness of the system can be guaranteed by setting the controller structure.

If Equation (17) of the corresponding system is a third-order system, according to the Routh stability criterion, the stability condition is $a_{2}a_{1}>a_{3}a_{0}$. According to the expression in formula (18), this is equivalent to requiring the stability index to satisfy $\gamma_{1}\gamma_{2}>1$. Similarly, the stability conditions of the fourth-order system are $a_{2}>\left(a_{1}/a_{3}\right)a_{4}+\left(a_{3}/a_{1}\right)a_{0}$ and $\gamma_{2}>{\gamma_{2}}^{*}$ . For the fifth-order and above systems, Lyapunov gives several sufficient conditions for different forms of stability and instability, among which the conditions suitable for the CDM are as follows [23]: if all the fourth-order polynomials of the system are stable and have a margin of 1.12 times, the system is stable. If some third-order polynomials in the system are unstable, the system is unstable. The stability conditions of the system can be described as :

(19) $$\begin{array}{c} \left\{\begin{array}{c} a_{i}>1.12\left(\frac{a_{i-1}}{a_{i+1}}a_{i+2}+\frac{a_{i+1}}{a_{i-1}}a_{i-2}\right),\\ \gamma_{i}>1.12{\gamma_{i}}^{*}\begin{array}{cc} , & i=2∼(n-1). \end{array} \end{array}\right. \end{array}$$

Manabe has proved that the system can obtain better stability and response characteristics when $\gamma_{i}>1.12{\gamma_{i}}^{*}$ and $\gamma_{i}$’ values are between 1 and 4. If the stability index is selected according to $\gamma_{i}>1.5{\gamma_{i}}^{*}$, the system’s robustness is improved by sacrificing stability and response characteristics [23]. With the help of some design experience, designers can consider stability, response characteristics and robustness by reasonably selecting the structure and parameters of the controller.

In this article, we use the stability index $\gamma_{i}$ values in the Manabe standard form. According to the Manabe standard form, the stability index $\gamma_{i}$ is defined as:

(20) $$\begin{array}{c} \begin{array}{c} \gamma_{1}=2.5,\;\;\gamma_{0}=\gamma_{n}=\infty ,\;\;\gamma_{i}=2;\;\;i=2∼\left(n-1\right). \end{array} \end{array}$$

Using the equivalent time constant τ and the stability index $\gamma_{i}$, the characteristic polynomial P(s) can be obtained as follows:

(21) $$\begin{array}{c} P\left(s\right)=a_{0}\left[\left\{\sum_{i=2}^{n}\left(\prod_{j=1}^{i-1}\frac{1}{\gamma_{i-j}^{i}}\right){\left(\tau s\right)}^{i}\right\}\;+\;\tau s\;+\;1\right]. \end{array}$$

By comparing the coefficients of the characteristic polynomial Equations (17) and (21), the CDM controller parameters can be obtained.

### 3.2. Measurement Noise Rejection

**Figure 5:**
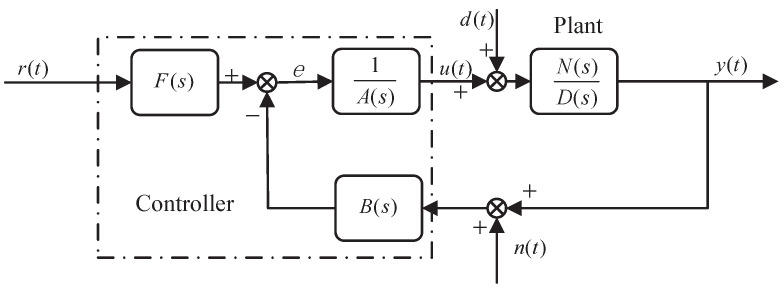
Basic structure of a CDM controller.

To meet with the design needs of real-life, we analyze the output effect of the measurement noise in the controlled variable *u* in Section 3.2. Usually the block diagram presented in Figure 4 is extended by including measuring noise. Measurement noise may have a different character, but it is typically dominated by high frequencies, and low-frequency noise would correspond to drift. High-frequency noise can be suppressed by limiting the bandwidth of the closed-loop system. CDM can restrain the influence of high-frequency noise by selecting the equivalent time constant τ to limit the bandwidth of the closed-loop system. The reason is that the rapid response of the closed-loop system is proportional to the bandwidth, The equivalent time constant τ is closely related to the setting time and bandwidth, which determines the rapid response of the system. Here, we give the analysis of low-frequency noise suppression. Those signals are represented in Figure 5.

The newly added signal n(t) denoting the measurement noise. We assume n(t) is bounded with $\left|n(t)\right|\leq \mu \cdot h\left(t\right)$, where μ and h(t) are a positive constant and a step-type signal, respectively [24]. In order to analyze the output effect of the measurement noise in the controlled variable u(t), the reference signal r(t) and disturbance signal d(t) is set to zero. This leads to a relationship between n(t) and u(t), n(t) and y(t) given by the following differential equation:

(22) $$\begin{array}{c} \begin{array}{c} -n\left(s\right)\cdot B\left(s\right)\cdot D\left(s\right)=\left(A(s)\cdot D(s)+N(s)\cdot B(s)\right)\cdot U\left(s\right),\\ -n\left(s\right)\cdot B\left(s\right)\cdot N\left(s\right)=\left(A(s)\cdot D(s)+N(s)\cdot B(s)\right)\cdot Y\left(s\right), \end{array} \end{array}$$

where n(s) is the Laplace transforms of n(t), U(s) is the Laplace transforms of u(t), Y(s) is the Laplace transform of y(t).

Let us impose $b_{i}\neq 0$ for i=0,⋯,m ,$d_{i}\neq 0$ for i=0,⋯,n in Equation (14). The product B(s)·N(s) is a q+m order polynomial and the product B(s)·D(s) is a q+n order polynomial, respectively. The polynomials will be denoted by C(s) and E(s) defined as:

(23) $$\begin{array}{c} \begin{array}{c} C\left(s\right)=\sum_{i=0}^{q+m}g_{i}^{\prime }\cdot s^{i},\;\;E\left(s\right)=\sum_{i=0}^{q+n}{g^{\prime \prime }}_{i}\cdot s^{i}. \end{array} \end{array}$$

Assuming r(t)=0,d(t)=0, this steady-state system behaviour will be easily handled in the Laplace domain. By applying the final value theorem, the following equality should hold:

(24) $$\begin{array}{c} \lim_{t\to \infty }y\left(t\right)=\lim_{s\to 0}s\cdot Y\left(s\right), \end{array}$$

However, in order to satisfy this equality, all the Y(s) poles must have negative real parts and no more than one pole can be at the origin [25].

Assuming causality and zero initial conditions, the application of Laplace transform to (22) leads to,

(25) $$\begin{array}{c} \begin{array}{c} U\left(s\right)=\frac{-E(s)}{A(s)\cdot D(s)+C(s)\cdot B(s)}n\left(s\right).\\ Y\left(s\right)=\frac{-C(s)}{A(s)\cdot D(s)+C(s)\cdot B(s)}n\left(s\right). \end{array} \end{array}$$

Applying the final value theorem to the above expression then,

(26) $$\begin{array}{c} \lim_{s\to 0}s\cdot -C\left(s\right)\cdot n\left(s\right)=0 \end{array}$$

Due to $\left|n(t)\right|\leq \mu \cdot h\left(t\right)$ and h(t), the Laplace transform is $\frac{1}{s}$, and we use $\frac{\mu }{s}$ replace n(s), thus expression (26) takes the following format:

(27) $$\begin{array}{c} \lim_{s\to 0}s\cdot -C\left(s\right)\cdot \frac{\mu }{s}=-\mu \cdot g_{0}. \end{array}$$

Since $g_{0}$ is equal to the product of $b_{0}$ and $k_{0}$ and since $b_{0}\neq 0$ then, in order for $g_{0}$ to be zero, the controller coefficient $k_{0}$ must be equal to zero. Similarly, when the controller coefficient $k_{0}$ is equal to 0, the measurement noise has no effect on the control *u*(*t*). Therefore, when the controller coefficient $k_{0}$ is equal to zero, the measurement noise does not affect performance.

## 4. Overall Design Ideas

**Figure 6:**
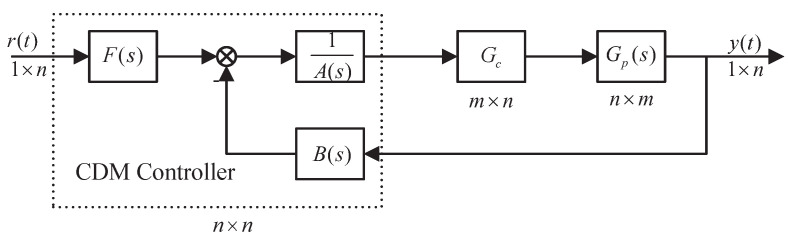
MIMO system control block diagram.

This paper designs a compensator $G_{c}$ and a centralized CDM controller for the n×m MIMO system in Figure 6. Systematic design ideas ensure the feasibility of decoupling design methods in large and small systems. At the same time, when the MIMO system interaction is minimized with high accuracy, the controller can achieve good control effects due to the robustness of the CDM. The most considerable advantages of CDM can be listed as follows:

1. A characteristic polynomial and controller are simultaneously designed. The characteristic polynomial specifies stability and response. The structure of the controller guarantees robustness. Thus, a simple controller, which satisfies the stability, response, and robustness requirements, can be designed with ease.
2. Compared with PID control that needed to develop different tuning methods for the process with various properties, it is sufficient to use a single design procedure in the CDM technique. This is an outstanding advantage.

The decoupling control and CDM controller design for the MIMO system can be summarized as the following steps.

**Figure 7:**
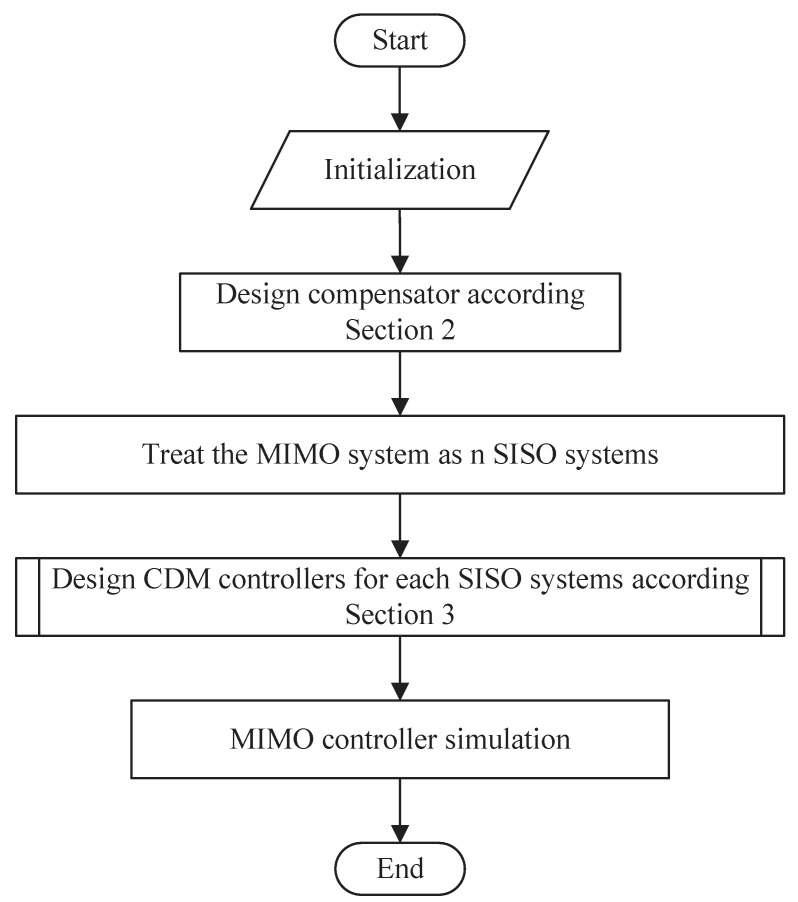
MIMO system control block diagram.

Figure 7 shows the design steps for MIMO systems, where the design process of CDM controllers is shown as the following:

1. Set the SISO controller parameters A(s) and B(s). $k_{0}$ = 0 is a good choice for measurement noise suppression.
2. Select CDM design parameters. The stability index $\gamma_{i}$ in this paper is in the Manabe standard form of Equation (20). As long as the value of the equivalent time constant τ is determined, the controller parameters can be obtained. The τ value mainly determines the response time of the system. Generally, the τ value is determined according to the design requirements of the system setting time and bandwidth.
3. Solve the SISO controller parameters. A(s), B(s) can be obtained by Equations (17) and (21). F(s) can be obtained by Equation (16).

**Remark** **2.** *The necessary condition for designing CDM controllers is that both denominators and molecules of the transfer function of the controlled object need to be expressed by rational polynomials. If there is a delay element in the transfer function of the controlled object, the improved Padé approximation method in reference [26] is used to deal with the delay element. According to the results of [26], the third-order improved Padé approximation is:*

(28) $$\begin{array}{c} e^{-sL}=\frac{60-24sL+3{\left(sL\right)}^{2}}{60+36sL+9{\left(sL\right)}^{2}+{\left(sL\right)}^{3}}. \end{array}$$

*where L is delay time.*

## 5. Simulation Experiment

This section conducts simulation experiments on four unique control targets to prove the effectiveness of this method. The experiments are evaluated with a step response of 1. The state variable is set to $x_{i}\left(i=1,\;2,\;3,\cdots ,n\right)$, and the system output is set to $y_{i}\left(i=1,\;2,\;3,\cdots ,m\right)$. Use the compensator for decoupling. When the interaction of the MIMO system is minimized, treat it as *n* SISO systems, and set each SISO system as $A_{i}=\left(i=1,\;2,\;3,\cdots ,m\right)$.

**Example** **2.** *Consider the two-input two-output second-order inertial system (sugar factory model) in [27]. The transfer function is:*

(29) $$G_{p}=\left[\begin{array}{cc} \frac{0.28}{21s^{2}+10s+1} & \frac{-0.33}{30s^{2}+11s+1}\;\\ \frac{0.4}{270s^{2}+39s+1} & \frac{0.5}{432s^{2}+42s+1} \end{array}\right].$$

**Figure 8:**
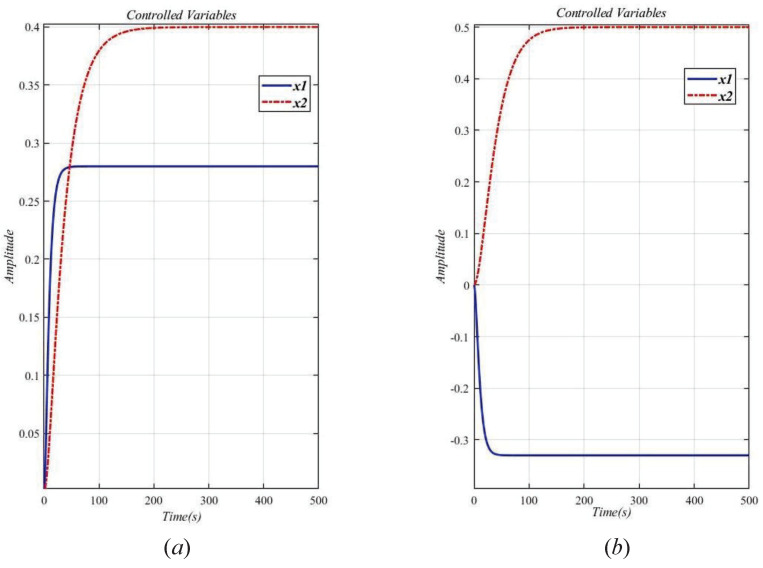
Step response curve of the original system (29) without decoupling design. (**a**) $x_{1}\neq 0$; (**b**) $x_{2}\neq 0$.

Without the decoupling design, the step response curve is shown in Figure 8.

It can be seen from Figure 8a that $x_{1}\neq 0$. As shown in Figure 8b, $x_{2}\neq 0$. The two loops are obviously related, so the system (29) is a related system to the interaction. Therefore a decoupling design method is used to eliminate the interaction of the original system.

Select the angular frequency $\omega_{0}=0.13$, and obtain the compensator as Equation (30).

(30) $$\begin{array}{ccc} & & G_{c}=\left[\begin{array}{cccc} 0.7807 & & 0.7606 & \\ -0.6256 & & 0.6507 \end{array}\right]. \end{array}$$

**Figure 9:**
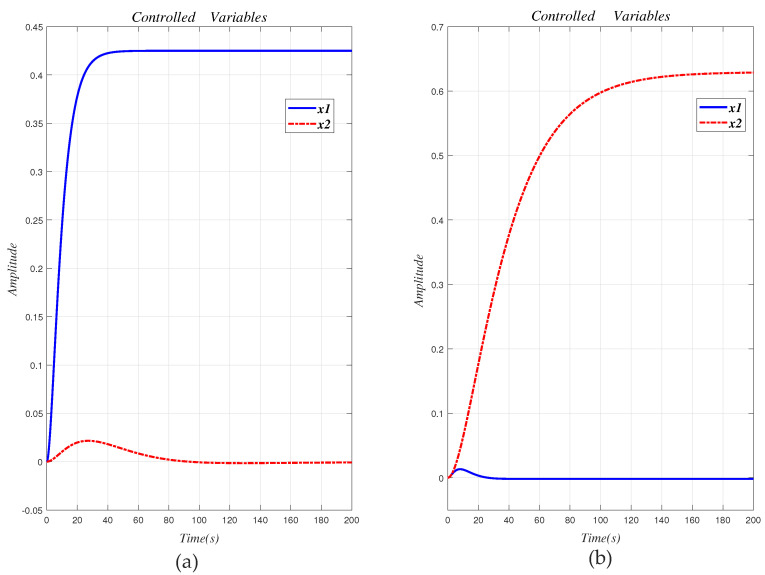
Step response curve of the original system (29) with decoupling design. (**a**) $A_{1}$; (**b**) $A_{2}$.

Apply the compensator (30) to the original system (29), then the decoupling system Q(s) is obtained. Figure 9 draws the step response curve of the original system (29) after the decoupling design. As can be seen from Figure 9, the interaction of Q(s) is effectively suppressed, especially in the static response part of the system. However, there is still a weak interaction in the dynamic response part. Overall, the decoupling effect is good.

The two SISO systems after decoupling are set to $A_{1}$ and $A_{2}$. For $A_{1}$ and $A_{2}$, use the stability index $\gamma_{i}$ and equivalent time constant τ in Table 4 to calculate CDM control polynomial parameters. Table 4 shows the equivalent time constant τ and CDM control polynomial parameter values.

**Figure 10:**
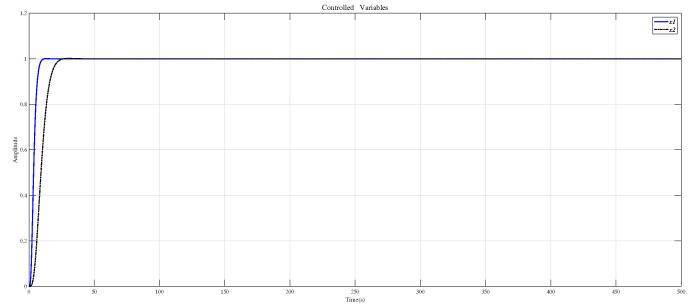
CDM controls $A_{1}$ and $A_{2}$ step response.

Using the CDM control polynomial parameters in Table 4 to control $A_{1}$ and $A_{2}$, the results are shown in Figure 10.

**Figure 11:**
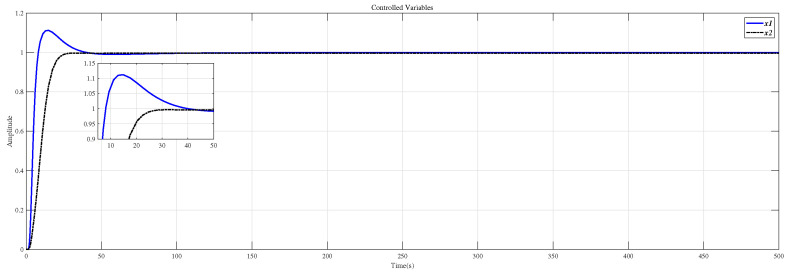
Use this method to control the MIMO system (29) step response.

Figure 11 shows the result of inserting the compensator (30) in front of the controlled object (29) and using the CDM parameters in Table 4 for control. Affected by the interaction of the dynamic response part, the system overshoot increases.

Here, the compensator (31) designed in [27] is compared with the method in this paper. Table 5 summarizes the results of evaluating the compensators (30) and (31) using formula (12). Table 5 shows that the decoupling effect of the compensator designed by the method in this paper is better.

(31) $$\begin{array}{ccc} & & G_{c}=\left[\begin{array}{cccc} 0.174 & & 0.479 & \\ -0.219 & & 0.503 \end{array}\right]. \end{array}$$

**Figure 12:**
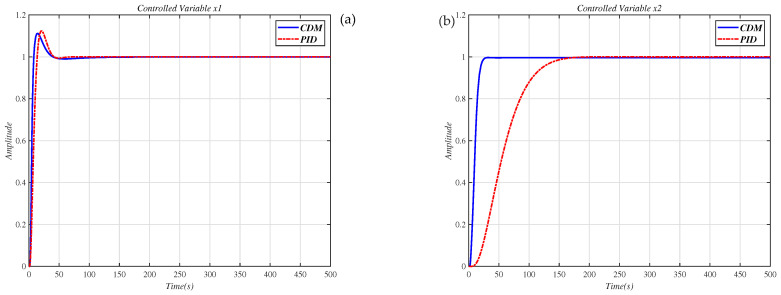
Use the PID controller in [27] and CDM controller to control the decoupling system Q(s) step response. (**a**) controlled variable $x_{1}$; (**b**) controlled variable $x_{2}$.

In [27], Masaya et al. designed a PID controller according to Shunji’s optimization method. We also use the design method proposed in [27] to design the controller for the decoupling system Q(s), which uses the compensator (30), and Figure 12 shows the results of the Masaya-PID controller and the CDM controller in this paper to control the decoupling system Q(s). From this figure and the performance values appearing in Table 6, it is seen that the CDM controller has a more successful time-domain performance.

**Figure 13:**
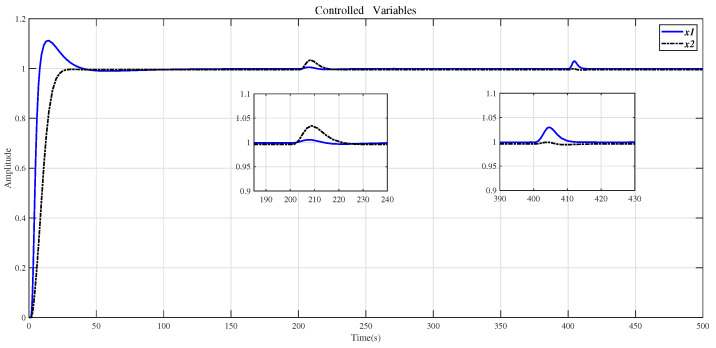
Use the method in this paper to control the step response of the original system (29) with the step disturbance.

**Figure 14:**
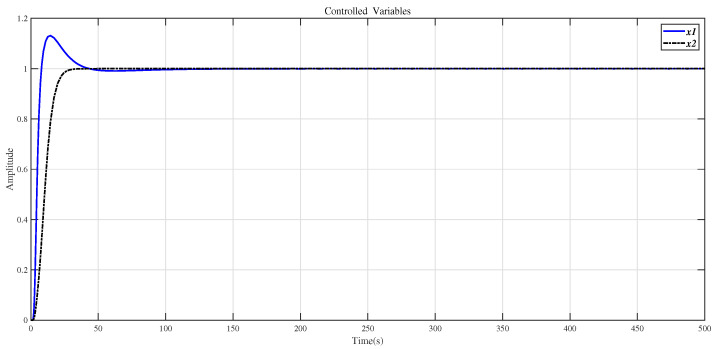
Use the method of this article to control the step response of the system (32).

In order to verify the robustness of the method in this paper, the system with disturbances and modeling errors is simulated. When there is a step disturbance in the original system, the control result is shown in Figure 13. According to Figure 13, the influence of the disturbance signal subsides in a short time. Suppose that the correct system model is represented by Equation (32), and the system model with errors is represented by Equation (29). The changes in the parameter of Equation (29) are in the interval ±15%. The compensator (30) is applied to the correct system model (32), and the CDM controller is used to control. The experimental results are shown in Figure 14. From the results in Figure 14, it can be seen that when the model has measurement errors, the control effect of the method in this paper is good. Figure 13 and Figure 14 show that the method proposed in this paper is robust.

(32) $$\begin{array}{c} G_{P}=\left[\begin{array}{c} \begin{array}{cc} \frac{0.238}{21s^{2}+10s+1} & \frac{-0.3795}{30s^{2}+11s+1} \end{array}\;\\ \begin{array}{cc} \frac{0.34}{270s^{2}+39s+1} & \frac{0.575}{432s^{2}+42s+1} \end{array} \end{array}\right]. \end{array}$$

**Example** **3.** *The multivariable four-tank system has a tunable transmission of zero [28,29]. With appropriate "tuning", this system will exhibit nonminimum-phase characteristics. Applying the nominal operating parameters given in [28,29] yields the four-tank system model:*

(33) $$G_{p}=\left[\begin{array}{cc} \frac{0.1987}{65s+1} & \frac{-0.3779}{\left(65s+1)(34s+1\right)}\;\\ \frac{0.4637}{\left(54s+1)(45.3s+1\right)} & \frac{0.16194}{54s+1} \end{array}\right].$$

For the four-tank system of the controlled object (33), the frequency $\omega_{0}=0.34$ is selected, and the precompensator is obtained.

(34) $$\begin{array}{ccc} & & G_{c}=\left[\begin{array}{cccc} -0.3262 & & 0.8884 & \\ 0.9455 & & -0.4597 \end{array}\right]. \end{array}$$

Use the evaluation formula (12) to evaluate the decoupling system Q(s) after the compensator formula (34) acts on the controlled object formula (33), and the result is 0.000013. It shows that after the decoupling design, the interaction of the controlled object (33) is weak, and the decoupling effect is well.

The two SISO systems after decoupling are set to $A_{1}$ and $A_{2}$. In order to suppress measurement noise, we select the controller coefficient $k_{0}=0$. Then, use the stability index $\gamma_{i}$ and equivalent time constant τ in Table 7 to calculate CDM control polynomial parameters. Table 7 shows the equivalent time constant τ and CDM control polynomial parameter values.

**Figure 15:**
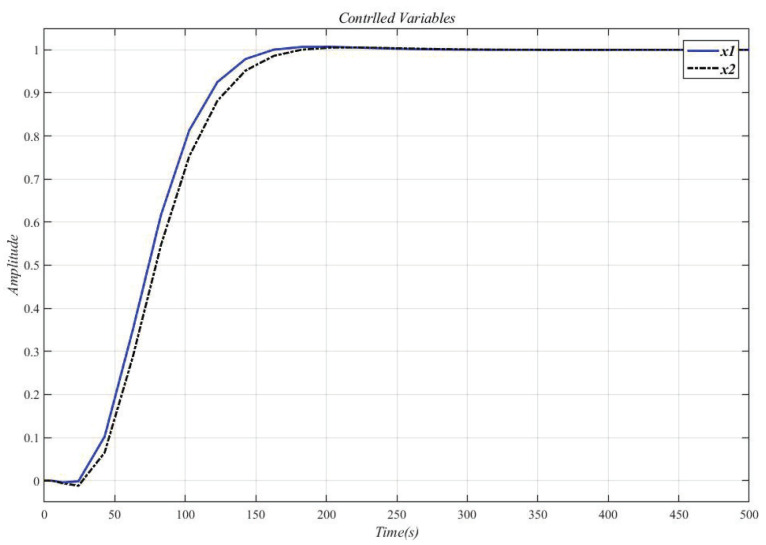
CDM controller controls $A_{1}$ and $A_{2}$ step response.

Use the CDM controller in Table 7 to control $A_{1}$ and $A_{2}$, the result is shown in Figure 15.

**Figure 16:**
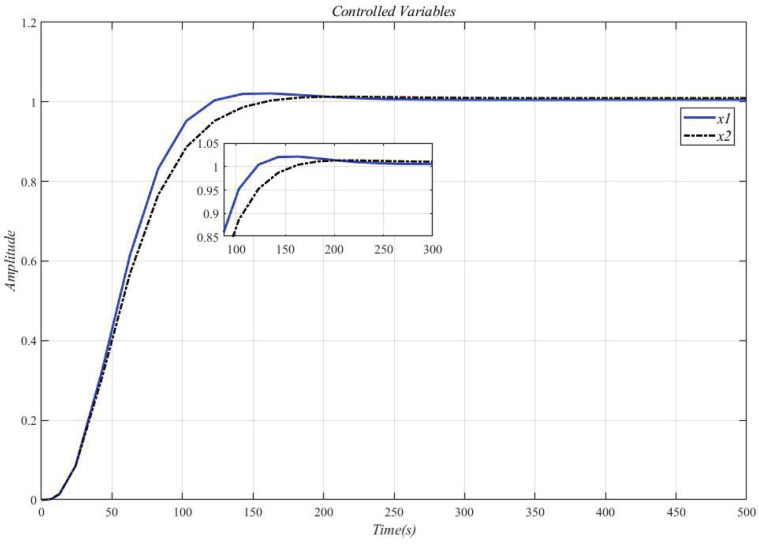
Use this method to control the MIMO system (33) step response.

Figure 16 shows the result of inserting the compensator (34) in front of the controlled object (33) and using the CDM parameters in Table 7 for control. It can be seen that the system overshoot is slightly increased due to the interaction.

**Figure 17:**
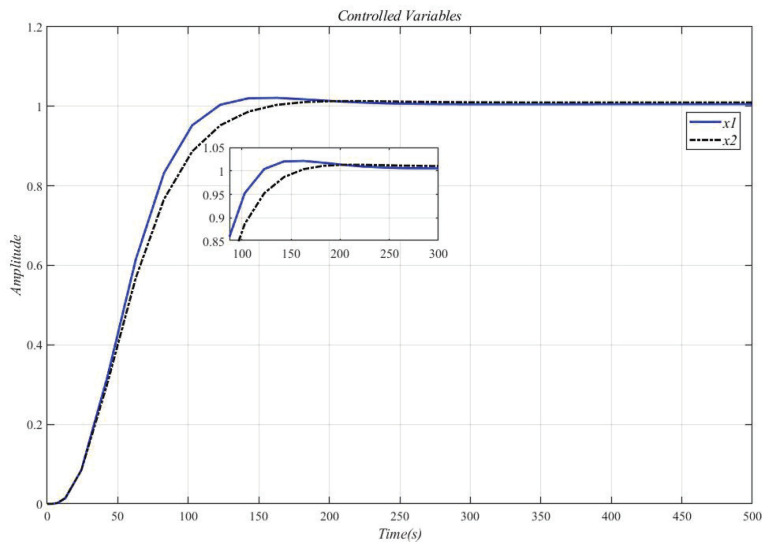
Controlled MIMO system (33) under the measurement noise.

Figure 17 shows the result of the controlled MIMO system (33) under the measurement noise whose magnitude is limited within [−0.0016, 0.0016]. It can be seen that the system response has not changed, and the measurement noise does not affect performance.

**Example** **4.** *The controlled object (29) increases the delay link to become the accused object (35).*

(35) $$\begin{array}{c} G_{P}=\left[\begin{array}{c} \begin{array}{cc} \frac{0.28}{21s^{2}+10s+1}e^{-0.71s} & \frac{-0.33}{30s^{2}+11s+1}e^{-2.24s} \end{array}\;\\ \begin{array}{cc} \frac{0.4}{270s^{2}+39s+1}e^{-0.59s} & \frac{0.5}{432s^{2}+42s+1}e^{-0.68s} \end{array} \end{array}\right]. \end{array}$$

Using the method in this paper, select the angular frequency $\omega_{0}=0.28$ and obtain the compensator.

(36) $$\begin{array}{ccc} & & G_{c}=\left[\begin{array}{cccc} 0.7809 & & 0.7625 & \\ -0.6247 & & 0.6470 \end{array}\right]. \end{array}$$

Use Equation (12) to evaluate the effect of the compensator (Equation (36)) on the controlled object (Equation (35)) to obtain the decoupling system Q(s), and the result is 0.000000057. It shows that the system interaction effect of using compensator decoupling is minimal, and the decoupling effect is good.

The two SISO systems after decoupling are set to $A_{1}$ and $A_{2}$. For $A_{1}$ and $A_{2}$, the delay link is approximated by the improved Padé approximation method in [26]. Then use the stability index $\gamma_{i}$ and Table 8 equivalent time constant τ to calculate the CDM control polynomial parameters. Table 8 shows the equivalent time constant τ and CDM control polynomial parameter values.

**Figure 18:**
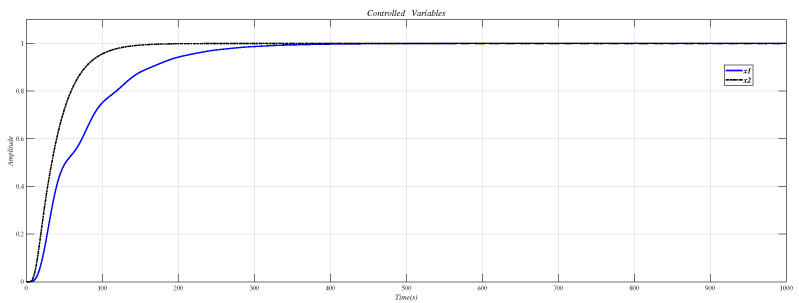
CDM control $A_{1}$ and $A_{2}$ step response.

Using the CDM control polynomial parameters in Table 8 to control $A_{1}$ and $A_{2}$, the results are shown in Figure 18.

**Figure 19:**
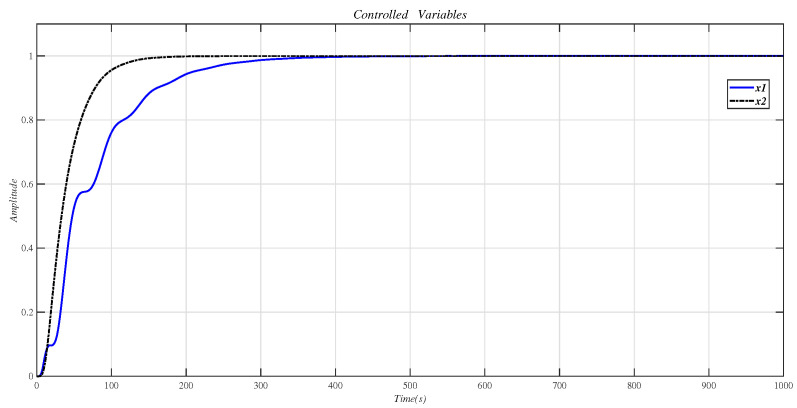
Use this method to control the MIMO system (33) step response.

Figure 19 is the result of inserting the compensator (36) before the controlled object (35) and using the CDM parameters in Table 8 for control. Figure 18 is the same as Figure 19, which proves that the decoupling effect is well.

**Example** **5.** *The controlled object (29) adds a line of input and a column output to become the controlled object (37).*

(37) $$\begin{array}{c} G_{p}=\left[\begin{array}{ccc} \frac{0.28}{21s^{2}+10s+1} & \frac{-0.33}{30s^{2}+11s+1} & \frac{0.38}{45s^{2}+12s+1}\;\\ \frac{0.4}{270s^{2}+39s+1} & \frac{0.5}{432s^{2}+42s+1} & \frac{0.6}{543s^{2}+68s+1}\;\\ \frac{0.9}{500s^{2}+30s+1} & \frac{0.45}{440s^{2}+45s+1} & \frac{1}{600s^{2}+89s+1} \end{array}\right]. \end{array}$$

Using the method in this paper, select the angular frequency $\omega_{0}=0.13$, and obtain the compensator:

(38) $$\begin{array}{ccc} & & G_{c}=\left[\begin{array}{ccccc} 0.6251 & & -0.7607 & & 0.8234\\ 0.3317 & & 0.0941 & & 0.0350\\ -0.7061 & & 0.6423 & & -0.5667 \end{array}\right]. \end{array}$$

Use the evaluation (12) to evaluate the decoupling system Q(s) after the compensator formula (38) acts on the controlled object formula (37), and the result is 0.0005411. The two SISO systems after decoupling are set to $A_{1}$, $A_{2}$ and $A_{3}$. For $A_{1}$, $A_{2}$ and $A_{3}$, use the stability index $\gamma_{i}$ and Table 9 equivalent time constant τ to calculate the CDM control polynomial parameters. Table 9 shows the equivalent time constant τ and CDM control polynomial parameter values.

**Figure 20:**
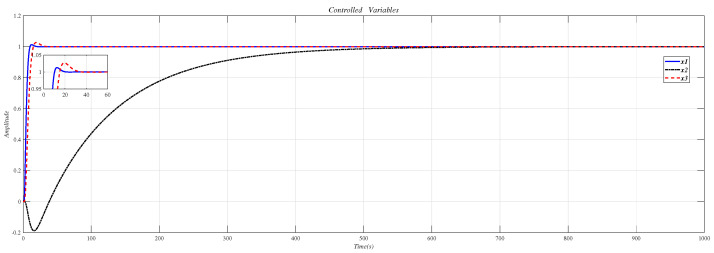
CDM control $A_{1}$, $A_{2}$ and $A_{3}$ step response.

Using the CDM control polynomial parameters in Table 9 to control $A_{1}$, $A_{2}$ and $A_{3}$, the results are shown in Figure 20.

**Figure 21:**
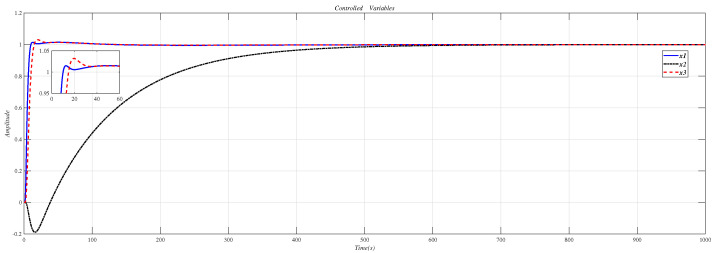
Use this method to control the MIMO system (37) step response.

Figure 21 is the result of inserting the compensator (38) before the controlled object (37) and using the CDM parameters in Table 9 for control.

## 6. Conclusions

This paper proposes a multivariable system controller design method based on the CDM and analyzes the controller’s suppression effect on measurement noise based on the CDM. The decoupling design is realized by designing the compensator in the frequency domain, and the compensator parameters are optimized through PSO. At the same time, use statistical tests to compare four evolutionary algorithms, including PSO, GA, SFLA, CS, to prove the advantages of PSO. After decoupling, the open-loop transfer function of the system is complex. Therefore, the controller structure design and parameter tuning are based on CDM. Finally, simulation experiments are carried out for four unique control targets. The results show that the decoupling effect of the MIMO system is good, and the designed system can take into account stability, response characteristics, and robustness at the same time, which confirms the effectiveness of the method.

## Figures and Tables

**Table 1:** The parameter settings of different evolutionary algorithms.

Evolutionary Algorithms	Parameter Settings
Genetic Algorithm (GA)	Population size = 50
	The times of iterations = 100
	Crossover probability = 0.9
	Mutation probability = 0.1
Shuffled Frog Leaping Algorithm (SFLA)	Population size = 50
	The times of iterations = 100
	Moving maximum distance = 0.02
Cuck Search (CS)	Population size = 50
	The times of iterations = 100
	Maximum discovery probability = 0.05
Particle Swarm Optimization (PSO)	Population size = 50
	The times of iterations = 100
	The weight of inertia = 0.35
	The self-learning factor = 1.5
	The population-learning factor=2.5

**Table 2:** Statistical results of different algorithms.

	Fmax	Fmin	Fave	Fstd	Time (s)
GA	0.8885	−0.2030	0.0859	0.2911	0.2099
SFLA	3.9953	−0.2095	0.5383	1.1147	0.2389
CS	0.1941	−0.2124	−0.1844	0.0845	0.1347
PSO	−0.1908	−0.2128	−0.2117	0.0039	0.0756

**Table 3:** The ranks achieved by Friedman, Friedman aligned and Quade tests.

	Friedman Ranks	Friedman Aligned Ranks	Quade Ranks
GA	1.9	7.32	1.81
SFLA	2.5	10.7	2.41
CS	1.2	5.6	1.38
PSO	1.1	4.5	1.10

**Table 4:** The equivalent time constant τ and CDM control polynomial parameter values.

	System *A*_1_	System *A*_2_
τ	11.2	16
F(s)	1.6811	0.8358
A(s)	$0.0313s^{2}+0.0855s$	$0.0921s^{2}+0.0928s$
B(s)	$\begin{array}{c} 6.9322s^{2}+6.1273s+1.681 \end{array}$	$\begin{array}{c} 25.3384s^{2}+7.9861s+0.83576 \end{array}$

**Table 5:** Comparison of evaluation.

	Ref. [27]	Proposed Method
evaluating value	0.048587	0.0000067

**Table 6:** Performance values of the time response curves shown in Figure 12.

	Settling Time	Max Overshoot %
Masaya-$y_{1}$	43	15
CDM-$y_{1}$	41	13
Masaya-$y_{2}$	172	1.5
CDM-$y_{2}$	26	0

**Table 7:** The equivalent time constant τ and CDM control polynomial parameter values.

	System *A*_1_	System *A*_2_
τ	120	100
F(s)	3.0833	2.8187
A(s)	$69.2884s^{2}+4.1572s+0.9019$	$75.3466s^{2}+3.8905s+0.9513$
B(s)	$-3231.1s^{2}-109.6204s$	$-3056.7s^{2}-88.0734s$

**Table 8:** The equivalent time constant τ and CDM control polynomial parameter values.

	System *A*_1_	System *A*_2_
τ	76	64
F(s)	0.0017	0.00028
A(s)	$0.3665s^{2}-0.0016s$	$0.0015s^{2}+0.000555s$
B(s)	$\begin{array}{c} 0.2464s^{2}+0.1321s+0.0017 \end{array}$	$\begin{array}{c} 0.0728s^{2}+0.0102s+0.0002773 \end{array}$

**Table 9:** The equivalent time constant τ and CDM control polynomial parameter values.

	System *A*_1_	System *A*_2_	System *A*_3_
τ	38.67	100	68
F(s)	−5.4186	3.6895	0.5582
A(s)	$0.0011s^{2}+0.0169s$	$\begin{array}{c} 0.0253s^{3}+0.08397s^{2}+12.9567s \end{array}$	$0.0027s^{2}+0.0055s$
B(s)	$\begin{array}{c} -25.92s^{2}-19.0082s\\ -5.4186 \end{array}$	$\begin{array}{c} 41568.98s^{3}+8205.15s^{2}\\ +447.8735s+3.6895 \end{array}$	$\begin{array}{c} 9.8979s^{2}+3.4319s\\ +0.5582 \end{array}$

## Data Availability

Not applicable.
